# Diapers in War Zones: Ethnomedical Factors in Acute Childhood Gastroenteritis in Peshawar, Pakistan

**DOI:** 10.1371/journal.pone.0119069

**Published:** 2015-03-13

**Authors:** Saira H. Zaidi, Carolyn Smith-Morris

**Affiliations:** 1 Department of Infectious Diseases and Vaccinology, University of California, School of Public Health, Berkeley, California, United States of America; 2 Department of Anthropology, Southern Methodist University, Dallas, Texas, United States of America; Leibniz Institute for Prevention Research and Epidemiology (BIPS), GERMANY

## Abstract

This article considers ethnomedical knowledge and practices among parents related to contraction of acute gastroenteritis among children in Peshawar, Pakistan. Research methods included analysis of the Emergency Pediatric Services’ admission register, a structured interview administered to 47 parents of patients seen in the Khyber Medical College Teaching Hospital, semi-structured interviews of 12 staff, and four home visits among families with children treated at the hospital. The use of native research assistants and participant observation contributed to the reliability of the findings, though the ethnographic, home-visit sample is small. Our research indicated that infection rates are exacerbated in homes through two culturally salient practices and one socioeconomic condition. Various misconceptions propagate the recurrence or perserverance of acute gastroenteritis including assumptions about teething leading to poor knowledge of disease etiology, rehydration solutions leading to increased severity of disease, and diaper usage leading to the spread of disease. In our Discussion, we suggest how hospital structures of authority and gender hierarchy may impact hospital interactions, the flow of information, and its respective importance to the patient’s parents leading to possible propagation of disease. These ethnographic data offer a relatively brief but targeted course of action to improve the effectiveness of prevention and treatment efforts.

## Introduction

Acute diarrheal disease in children is a major concern in both the developing world and politically violent contexts. The social and economic circumstances that normally create food and water insecurity are exacerbated in these areas by weakened medical infrastructures and limited resources, producing epidemic levels of disease in the already vulnerable population of children under the age of 5. Even where biomedical intervention and education is present, transmission rates can remain high because of diverse cultural and socioeconomic factors, as was recently discussed by Nurul Huda [1]. The difficulty of reducing diarrheal infection through biomedical interventions alone highlights the need to complement medical education and oral rehydration therapies with culturally-informed strategies and community awareness. As Vecchiato, Zwane and Kremer demonstrated for tuberculosis control and diarrheal disease (respectively), anthropological research is useful for identifying the cultural correlates of disease, particularly those which determine patient utilization of available, proven prevention techniques [2,3].

We therefore conducted a brief ethnographic study to better understand the context of acute pediatric gastroenteritis in Peshawar, Pakistan, which is a major educational, political and business center of Khyber Pakhtunkhwa. It is a culturally diverse city near the border of Pakistan and Afghanistan—currently a dangerous site of terrorist violence and heavy refugee flows. This research illustrates the utility of ethnographic methods for understanding, even in short research periods, the impact of complex patient lifeworlds on willingness to follow biomedical advice.

### Background

Gastroenteritis, the inflammation of the gastrointestinal tract, is a well-known illness that plagues over 111 million children under the age of five worldwide annually of which 1.3 million die annually [4,5]. It often leads to dehydration and death. In low-income countries, acute gastroenteritis is the second leading cause of death among infants and children under the age of 5 [6]. Regional differences of global deaths due to diarrhea are large with proportionate mortality in Southeast Asia account for 31.3% of deaths [7]. Exposure to viral and bacterial infectious organisms occurs primarily through consumption of contaminated food and drink. Fecal to oral transmission is facilitated by unsanitary conditions. Prevention of transmission involves improvement of sanitary conditions, health education, and nutrition [8]. As such, anthropological research and quantitative methods are complementary in assessing the extent and reason behind diseases propagation, and in identifying effective strategies in socially complex and pluralistic health care settings [9,10,11]

Although the causal mechanisms of acute gastroenteritis have been well established, the contextual reasons for their occurrence in any given place vary. Factors include economics and poverty level, sanitation infrastructure, cultural and historical factors related to sanitation, and the behaviors and beliefs of patients [12]. Deprivation manifested by household crowding and low maternal education has also been identified as correlated to increased transmission [13]. Identified behavioral factors affecting transmission include sub-optimal breastfeeding duration or practices [14], storing food at room temperature rather than refrigerating, failure to wash hands, failure to dispose of feces hygienically, and drinking contaminated water [15].

In the research conducted by Mumtaz, Ahmed and Ali, the factors mentioned above apply to the Afghan refugee population, that lives in poor hygienic conditions and among large families. They report that children are “not properly looked after and hand washing is not practiced” [16]. Similar observations were made in the study conducted by Nagamani in Indonesia and Ghana [17]. They remind us that the increased infection rate in children under five years might also have been due to the immunologic immaturity of this age group, particularly those under the age of two years, through early weaning and contaminated food and water.

Hospitals and policy makers are developing strategies to encourage prevention, home treatments, appropriate health seeking, and treatment compliance. To this end, cultural and contextual information is needed to aid strategy development for this setting. This research is increasingly important to hospitals and other policy makers in Peshawar, which have been devastated by war, terrorist attack, and refugee influx since the Soviet War in Afghanistan in the 1980s. Such research must be responsive to local beliefs and events, including the constantly changing conditions of war as well as the multi-cultural aspects of refugee zones [18,19]. As such, we conducted a brief but multi-faceted ethnographic study to better understand the cultural and contextual factors that might influence transmission and treatment adherence for acute gastroenteritis among Peshawar children.

### Research Setting

Peshawar has not been formally studied for risk factors and prevalence of acute gastroenteritis. Crude estimates from the Hayatabad Medical Complex, a teaching hospital in Peshawar, indicate that acute gastroenteritis accounts for 25% of all admissions to the pediatric unit of the hospital in 2004 [20]. Our objective was to supplement these data with up-to-date contextual information and speak to factors leading to the prevalence of acute gastroenteritis.

**Fig 1:**
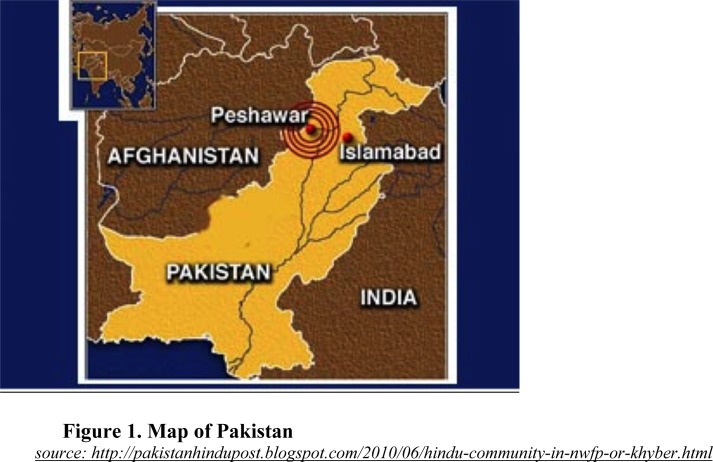
Map of Pakistan.

Located twenty miles from the Afghan border, Peshawar, Pakistan is filled with refugees that fled to Pakistan during the Soviet War of the 1980’s and the current war in Afghanistan (Fig. 1). The devastating floods of 2010 in Pakistan also displaced a large population of people that are now living in Peshawar. Peshawar is the provincial capital of the Khyber Pakhtunkhwa province of Pakistan. Its neighborhoods range from refugee encampments to urban dwellings. This unique and diverse population is of low socioeconomic status and comprises a significantly underserved area. There are biomedical, local, and government based health care resources, although biomedicine is broadly regarded as the most effective. This combination of factors made Peshawar an excellent site for understanding cultural and contextual factors in treatment and control of child gastroenteritis.

## Methods

Ethnographic research was conducted in Khyber Medical College Teaching Hospital, which serves as a central treatment facility for the Peshawar region. The Emergency Pediatric Services (EPS) and the Out-Patient Department (OPD) where all gastroenteritis patients are seen became the clinical home of the project. In Khyber Teaching Hospital, the OPD primarily focuses on chronic gastroenteritis patients: those with diarrhea for greater than 14 days. Patients who suffer from diarrhea for less than 14 days are diagnosed with acute gastroenteritis and are cared for in the EPS. These two wards offered important contrasting perspectives on the chronic versus acute forms of gastroenteritis, and resulting hospital admissions, in the region. Analysis of the EPS admission registry from June and December 2011 focused on the key words ‘diarrhea’ and ‘acute gastroenteritis’ (or ‘AGE’). All patients who had diarrhea or ‘AGE’ written under diagnosis was counted as an acute gastroenteritis patient.

All patients under the age of five years who came to the EPS with primary complaint of diarrhea between 10am and 3pm during the thirty-day study period were invited to participate in the study. Only those patients whose children were over the age of five years and did not have a primary complaint of diarrhea were excluded. Recruitment yielded a sample of 47 patients under the age of five whose accompanying parents completed a comprehensive, semi-structured interview and 52-item questionnaire.

Local research assistants were employed for ease of communication, including one local nurse who was recruited based on her Pashto language knowledge and trained on how to translate and interpret patient information. The nurses’ biases may include the summarization of patient communication and interpretation of disease descriptions. After consent procedures, all interviews were conducted in either Urdu or Pashto based on the patient’s preference and recorded using a handheld recorder so that parents could be invited to elaborate on their responses. Interviews were conducted in the EPS unit, OPD unit, or patient’s home and ranged from 15 to 45 minutes in length. Parents were given an antibacterial soap as a gift for their time. These gifts were considered an incentive to participate in the research and had the added benefit of emphasizing the importance of sanitation.

The questionnaire addressed patient demographics, ethnic and religious background, household characteristics, primary caregiver information, an overview of water and food resources, and an assessment of the parent’s knowledge of the disease. The objective of the questionnaire was three-pronged: to assess the patient’s background, possible underlying causes of disease propagation, and prior disease knowledge.

Questionnaire responses along with general observations were documented and later grouped into pertinent topics including sanitation, water usage, counseling, disease, expectations, hierarchy, socioeconomic causes, and cultural values. Interview responses were analyzed through grounded analysis and discussion of thematic content to consensus between both authors. Items mentioned by at least 5 different informants were considered thematic. The themes reported below were raised by at least 9 different informants and were, therefore, considered salient.

The structured interviews with parents were complemented by semi-structured interviews with doctors, nurses and staff members regarding water sanitation, hygiene, and the political atmosphere. A total of twelve staff in four different professional positions were interviewed. They were selected based on their involvement with diarrhea patients and their availability.

The specific circumstances and conditions in Peshawar at the time made this a challenging research site. The U.S. Department of State believes the situation to be sufficiently dangerous that it has discouraged American citizens from traveling to Pakistan. Ms. Zaidi was able to secure access due to her own status as a second generation Pakistani-American who is fluent in Urdu, has spent extensive time in Pakistan and is familiar with local customs and norms. Additionally, through previous work and personal connections, Ms. Zaidi had the support of a network of physicians and hospital administrators in Pakistan. Her knowledge of local customs and ability to quickly integrate based on her ethnic heritage were critical to secure access to patients, data and resources needed to make this project a success.

We took a number of additional steps to supplement Ms. Zaidi’s own knowledge and experience. First and foremost, Ms. Zaidi maintained daily contact with a university police officer (from her host university and IRB institution) and hired a local driver / security guard to help her navigate the neighborhoods of Peshawar safely. The three Peshawar research assistants were compensated at rates appropriate for their location and skill. They were primarily used to help interview participants and record data in the form of photographs of participants and some of their local water sources. Neutral probes and prompts were used in interviews, consistent with ethnographic interviewing techniques, and longer narratives were encouraged given the ethnographic nature of the research. Ms. Zaidi conducted home visits and unstructured interviews at four patients’ homes. There, patients and their female family members were questioned about their living environment, practices related to sanitation, water sources, and their explanatory models of acute gastroenteritis [21]. Ethnographic methods of immersing oneself in other cultures are important for the purpose of capturing full contextual detail from patients’ lifestyles, and is particularly important for cross-cultural work in which patients come from a multitude of ethnicities and backgrounds (as in refugee zones). These methods also allow for additional, alternative perspectives on patients’ lives for which simple, hospital-based questionnaires would be inadequate by themselves.

Finally, although our goal did not include a complete study of the refugee experience and context of violence, a note about these variables is necessary. During our research period alone, two bombs killed over 60 people, including a doctor from Khyber Medical College Teaching Hospital. Patient #40 lived in the neighborhood of Bara, where terrorists and extremists often gather. The patient’s mother complained that normal people could not leave their home for days at a time and were stuck in one room: “the diarrhea is due to unsanitary conditions, but Taliban (a fundamentalist Muslim political movement in Afghanistan) and police are around my house, I can’t leave”. Thus, participant observation allowed us to assess sanitation levels from an etic perspective and to observe the daily practices that might affect patient compliance with biomedical instructions, and ultimately the prevalence of acute gastroenteritis.

### Ethics Statement

This study was approved by the Institutional Review Board of Southern Methodist University and written informed consent was obtained from all subjects.

## Ethnographic Results: Cultural Factors Affecting Diarrheal Infections

From EPS records reviewed on site, approximately 200 patients are seen per day in the EPS, approximately 41% of whom came in with a primary complaint of acute gastroenteritis. Our sample of 47 patient families during a period of 30 days in the Winter of 2011 is non-representative, but offers insights into the beliefs, practices, and knowledge of this large patient group. Our ethnographic data point to three salient local practices in contraction of pediatric gastroenteritis. They are: (1) teething and its relationship to gastroenteritis, (2) the use of rehydration solutions, and (3) diaper usage.

### (1) Teething

Teething was described the emergence of new baby teeth and often a subsequent chewing of objects to alleviate pain. In Peshawar, eleven out of 47 parents questioned (23%) cited a link between acute gastroenteritis and teething. 9% cited a link without prompting, and an additional 7 parents (15%) cited a link when asked directly. This belief led parents to engage in certain measures or lack of oversight that might exacerbate, rather than ameliorate gastroenteritis, a problem that has been documented in several developing contexts. These actions include, for example, not actively preventing their children from putting contaminated foreign objects in their mouth. In a study of maternal beliefs concerning diarrhea in North India, more than 50% of 600 mothers blamed their children’s diarrhea on teething. Similar beliefs have been reported from other countries such as Nepal [22,23].

Notably, hospital staff members were also likely to affirm a relationship between teething and contraction of gastroenteritis. The head of the Malnutrition Unit at Khyber Teaching Hospital, explained “kids put their dirty hands and dirt in their mouth especially when teething, which causes them to contract acute gastroenteritis.” Conversely, a pediatric physician intern in the children’s ward—attributed causality not to dirt, but to the stress a child undergoes when his/her teeth first being to emerge. She believed that acute gastroenteritis contracted in this manner is self-limiting and resolves on its own within two to three days. Additionally, a nurse in the EPS department also believed teething causes acute gastroenteritis. Misconceptions or oversimplification on the part of parents of the causal pathways to AGE may be a result of interactions between physicians and parents. Physicians often asked parents if the child was teething, and immediately afterward wrote a prescription for medication to treat gastroenteritis without further investigation. The conflation of correlation and causation is not uncommon, and several nurses, doctors, and nutritionists, along with parents, cited teething as a cause of acute gastroenteritis.

We were particularly interested in how informants expressed the relationship between teething and acute gastroenteritis. Their explanations of the relationship spanned a wide range: no explanation; the stress or pain of teething (“the child is in a lot of pain”; and “[I] gave my other children oral drops that I put in their water to ease the teething pain so that they would not be in pain and have acute gastroenteritis”); the increased exposure to dirt or infectious agents associated with greater mobility at this age; or the increased exposure to dirt or infectious agents associated with putting hands and items in the mouth. Research from Macknin indicates that in actuality teething causes children pain, which leads to an increase in the number of foreign objects the child puts in his or her mouth [24]. While the mastication of these typically unhygienic objects often leads to acute gastroenteritis, the mere emergence of teeth in babies does not. Since even hospital staff can operate under these beliefs, it will be important that prevention efforts ascertain the explanatory models of parents and providers *as part of* prevention and intervention.

### (2) Rehydration Solutions

Rehydration solutions are essential in preventing dehydration that is the primary cause of death associated with diarrhea. Oral Rehydration Therapies, or ORT, can include cost effective medicine or dietary supplements that help manage acute gastroenteritis symptoms. Newer ORT’s fortified with Zinc are quite effective and are widely used in the region to shorten the duration of diarrhea [25]. Improper water to ORT sachet ratio along with unclean water, however, can delay recovery. Additionally, certain local approaches to rehydration worsen acute gastroenteritis.

Questionnaire responses and personal narratives in our research illustrated that there was a surprisingly high rate of tea and *sherbet*, a local sugary drink, usage in children. Seven out of 47 parents (~15%) cited feeding their children either tea or *sherbet* to remedy dehydration from acute gastroenteritis. One mother complained to the nurse “my child won’t drink any water when dehydrated” and so the nurse recommended adding lemon and sugar, or Tang, an orange flavored drink mix available in local stores, to make it tastier for the child.

*Chai* and *kava* are common hot beverages in Peshawar, Pakistan. *Kava* is a native green tea that is drunk throughout the day by all socioeconomic classes regardless of outdoor temperature. While teas are commonly recommended to patients sick with a cough and are used routinely around the world for medicinal purposes, they can actually be harmful in the case of acute gastroenteritis [26]. These local teas are infused with sugar that worsens the rate of dehydration [27]. A typical homemade oral rehydration solution consists of six teaspoons of sugar and ½ a teaspoon of salt in a liter of boiled water [28]. *Chai* and *kava* have anywhere from 1 teaspoon to 2 ½ teaspoons of sugar in. 25 liters (one cup).

Excessive amounts of sugar can make diarrhea worse and prevent intake of important minerals necessary for the child, whom is often dehydrated [29]. The same can be said about lemon and sugar water, as well as Tang flavored water. Tea or *chai* contains excessive concentrations of aldehyde (a carbon double bonded to oxygen, a hydrogen and an R group) and low concentrations of sodium. The inappropriate glucose-to-sodium ratio impairs water absorption, and the large osmotic load creates an osmotic diarrhea, further worsening the degree of dehydration [30]. These sugared drinks, if not properly made, can actually worsen acute gastroenteritis symptoms. Furthermore, tea has caffeine (a diuretic), which can also possibly worsen acute gastroenteritis [31].

**Fig 2:**
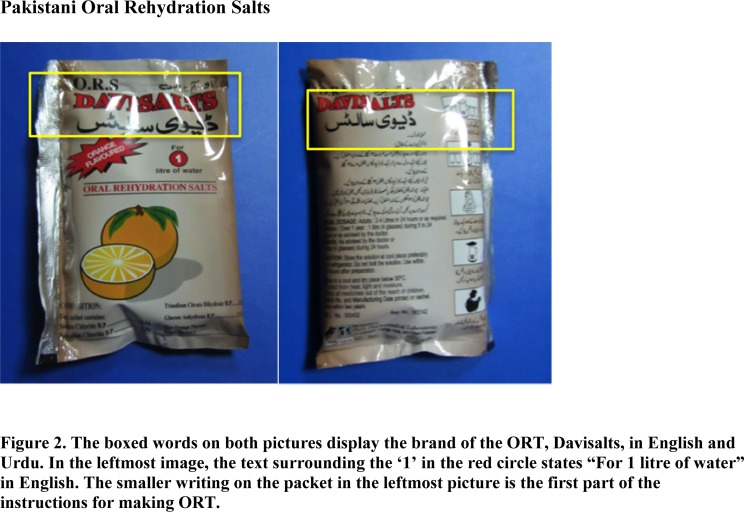
The boxed words on both pictures display the brand of the ORT, Davisalts in English and Urdu.

Communication of instructions for ORT use is an additional cause for concern. Many ORT medications are packets of medication, which must be prepared in a specific way; namely, mixed into a precise quantity of boiling water. The written instructions pose an issue for this population, as 75% of the respondents interviewed were illiterate (Table 1). Literacy levels were determined based on whether or not the mother could read and write as indicated by yes, only read or write (partial) and could not read nor write (no).These sachets are purchased over-the-counter as per the doctor’s instruction and have explicit directions on how to prepare the ORT on the back (Fig. 2). The patient’s family is told to purchase the ORT and is typically given a quick overview of the purpose of it and a times how to prepare it by the prescribing physician. Many women simply pour the packet into what they feel is a sufficient pot of water. Women who cannot read the instructions are limited to instructions that they receive orally. They are at a disadvantage because their compliance is dependent on their ability to remember these oral instructions. This likely also limits their access to any supplementary written explanation of the medication.

**Table 1:** Mother literacy levels.

Response	Number of Participants	Percentage
Yes	11	25%
Partially	1	2%
No	33	73%
No Response	2	-
Total	47	100%

The literacy issue was illustrated in the children’s ward one day, when patient #24’s mother was chastised by the doctor. She had yet again over-diluted her child’s baby formula, and therefore her child was not receiving sufficient nutrients. Staff members reported that over-dilution is a common problem not only with bottle feeding, but with various medications as well. Yet literacy is not the only problem in this scenario; poor methods of communication and impatience with differences in explanatory models due to lack of resources exacerbate illness through under-effective treatment efforts. Parents may misunderstand biological processes and hold incorrect perceptions about medication or treatment. Additionally, clinicians may fail to give information in a way that can be understood and utilized by parents, given the context of their lives, their literacy, and their resources.

### (3) Diaper Usage

Socioeconomic disparity was not assessed, but was manifest in parents’ varied access to nutritional foods, in sanitation, and in the use of diapers versus cloth or no diapers (Table 2). Store-bought diapers sufficiently contain child’s waste and minimize the risk of oro-fecal disease; however, they are expensive and cannot be reused like cloth diapers. Clothe diapers, while more economical, are unable to fully contain children’s waste without a protective external lining, increasing the likelihood of exposure to fecal material. Foregoing diapers altogether is least expensive, but also least likely to contain children’s waste.

**Table 2:** Diaper Usage.

Diaper Type	Number of Participants	Percentage
Store-bought	9	45%
Cloth	8	40%
No diaper use	3	15%
No Response	27	-
Total	47	100%

Acute gastroenteritis is spread via fecal-to-oral transmission as well as several alternative mechanisms [32]. In resource-poor settings, poor fecal containment and inadequate washing practices promote the quick spread and reinfection of children with acute gastroenteritis [33]. Household crowding increases the likelihood of exposure to unsanitary conditions and will be particularly problematic in households with young children [34]. Given the climate of violence in Peshawar today, crowded household conditions already common in the poorest areas are made worse by fear of gathered terrorists in the neighborhood.

To better understand parents’ contingent strategies in this atmosphere of violence, the clinical questionnaire was modified during the field work. Twenty of the 47 (43%) patient’s parents were asked to specify the type of diaper, if any, they used for their children. This sub-sample offers insight to not only socioeconomic factors in fecal to oral transmission, but also the household circumstances that impact spread and reinfection of acute gastroenteritis. Twelve out of the 20 or 60% of those patient’s parents interviewed said they used cloth or no diapers for their children. Two of those that used store-bought diapers acknowledged that they “didn’t have children for years and so now they are spoiled with store bought diapers”. Cloth diapers and clothing alone (no diaper) allow leakage of fecal material onto secondary surfaces such as bed sheets, the floor, or the hands of people caring for the child. This is particularly an issue when multiple children and parents sleep on the same surface

Use of cloth, disposable, or no diapers was explained here as an economic issue and a cultural factor or explanatory model difference. We acknowledge that certain contextual and economic factors are beyond the ability of clinicians to ameliorate, and possibly even address, including warzone and terrorist violence in neighborhoods. In these circumstances, acknowledgment of and sensitivity to these conditions can be helpful. At Khyber Teaching Hospital, for example, food supplements are given to patients in the Malnutrition Unit. Although disposable diapers would have been helpful in reducing fecal to oral transmission, they did not appear to be a central element of prevention or treatment resources.

## Discussion and Conclusion

Acute gastroenteritis is prevalent among children in Peshawar, Pakistan and has several cultural and contextual factors that contribute to transmission rates. This research suggests that themes of teething, rehydration strategies, diaper usage, and hierarchy will be important for future prevention and treatment. Local views about teething and its connection to acute gastroenteritis affect the actions that parents take to avoid transmission. Finally, lack of information about the proper and most effective use of rehydration solutions potentially worsens acute gastroenteritis symptoms, as does inadequate containment of children’s waste.

How might these ethnographic lessons be productively applied through local health care structures and authorities? Anthropologists and other social scientists have shown the value of in-depth, local knowledge for effective health interventions, regardless of the location [35, 36, 37]. But sites of conflict, danger, extreme resource pressure like refugee camps are particularly vulnerable to the challenges of conflicts in healing modalities or beliefs, limited access to health care, and over-taxed health care infrastructures and staff [38, 39]. Many examples may be found; Halvorson’s work on women’s role in making or breaking the “chain of [diarrhea] contamination” within the household sphere is informative in this regard [40]. Sustained changes in behavior as well as the enhancement of women’s capacity to solve health problems were found to be crucial to the objectives of rural water, sanitation and diarrheal disease control programs [41]. Caregivers frequently fail to recognize children’s diarrhea, especially among younger infants and when illness signs are less severe [42]. Mothers’ responses to diarrheal diseases are mitigated and shaped by community values, belief systems, gender dynamics, and the socioeconomic circumstances in which they live, work, and raise families [43]. Our research confirms that programs which take mother’s beliefs and resources into account are more likely to be locally relevant and sustainable, if not also successful. Halvorson’s gendered finding is a good example—although only one example—of the importance of ethnographic data to effective health care.

Peshawar is a patriarchal society in which the eldest male governs the household [44]. Women are the primary caretakers of children, and in the hospital only women were allowed inside the children’s ward, which Ms. Zaidi observed and noted through participant observation and personal narratives. Additionally, there is a distinct hierarchy among the hospital personnel, which impacts interactions, the flow of information, and its respective importance to the patient’s parents [45]. Women and their children primarily frequented the children’s wards in the hospital. On occasion, men would enter EPS with their children, primarily when speaking to the physician, but whenever the child had to be sent in to the EPS for injections or IV rehydration, men (even fathers) were not allowed to enter the EPS. That children’s hospital wards are the domain of women attests to the patriarchal nature of the Peshawarite gender roles. Women are universally the primary caregivers [46]. Men accompanied their women primarily for their protection and supervision, and not for their role in childcare. This local ethnographic and qualitative data, even acknowledging its small scale and sample, has provided inexpensive but crucial information about the social context of health care in Peshawar, including gender norms for home care, spending on medications, and hygiene.

But to ensure ethnographic findings are applied, the authoritative structures within health care systems must also be taken into account. There is usually a clear professional hierarchy in public hospitals such as Khyber Teaching Hospital. Doctors are at the top of this hierarchy, after which comes nurses and nutritionists followed by other personnel. This hierarchy is evident in the interactions between doctors and nurses, and in the interactions of both medical groups with patients. While there is often friendly banter and conversations between doctors and nurses, quite often one of the physicians would raise his voice at one of the nurses for not following his directions. A nurse in the EPS department at the beginning of data collection told Zaidi, “the doctor is being nice and putting on a good show for you, but he scolds us and the parents often”. Similarly, doctors and nurses berate their patients’ parents. When asked why there appeared to be a lack of patience with parents, a nurse explained, “One, many parents want their child to be cured right away. They do not comprehend that the process will take a while and they do not want to wait. Two, parents feel like they need injections and that they have not been properly treated until they receive antibiotics or injections”. These circumstances frustrate doctors and nurses who end up berating parents for their lack of understanding and knowledge. Yet despite the scolding and hierarchy, and regardless of how the patient and his or her parents felt about the doctor, they greatly valued the doctor’s opinion and followed their advice when feasible and properly understood. These social and hierarchical variables impact not only how care and education are delivered, but the ways in which patients can receive information and collaborate in health management.

Peshawar was chosen as a study site to highlight the impact of its unique context. Our research contributes data on regionally pertinent cultural beliefs and patterns of behavior relevant to the propagation of acute gastroenteritis. However, we also contribute to the growing body of literature that calls for collaboration between quantitative and clinical strategies, on the one hand, and qualitative studies of context, on the other. The widespread presence of teething explanations for diarrhea particularly held by practitioners who have significant authority in the community, indicates that these beliefs will not be easily dismissed. Preventive measures must be taken in Peshawar that simultaneously inform patients and their families about the infectious agents behind acute gastroenteritis *and* about the behavioral links to teething children. Parents and staff should be encouraged to understand the differences between causal relationships, and associative ones; but simultaneously, local structures of authority in health care must value and seek out qualitative forms of data from the social, political, and religious contexts. At the very least, prevention and intervention would respect that this differentiation is not universal. Pinpointing these simultaneous plausible causes of acute gastroenteritis in this patient population will allow for directed, well-informed initiatives to be taken. The study results were presented to the newly hired head of the hospital in hopes to strengthen in-house pediatric training directives. They await further discussion in published form to share among hospital staff.

As a pluralistic society, Pakistan often encompasses several ethnomedical systems, and there is evidence that biomedicine has an “incomplete” authority here, since even its practitioners have competing ideas of cause and effect [47]. Explanatory models of *both* parents and medical personnel are relevant to the prevention and treatment effort. They show important contrasts between lay and professional discourses, and suggest the most efficient targets for improving adherence and understanding in treatment.

We propose brief but intensive and holistic ethnographic strategies to assess the lived experience of patients in their home environments, and to understand the context of staff–patients relationships. Local systems of authority, knowledge sharing, gender hierarchy, the diversity of explanatory models across all actors, information about resource allocation within households, and how education and treatment interactions between staff and patients, are all necessary variables for informed patient care. While great strides have been made in the area of affordable medications, acute gastroenteritis remains the second leading cause of death in children under the age of five. Failure to take into account these complex social variables has distinctive impacts on the continuation of these epidemics. More holistic approaches to health care and education are a constructive way forward.
